# The Economic Impact of Smoke-Free Laws on Restaurants and Bars in 9 States

**DOI:** 10.5888/pcd10.120327

**Published:** 2013-08-01

**Authors:** Brett R. Loomis, Paul R. Shafer, Martijn van Hasselt

**Affiliations:** Author Affiliations: Paul R. Shafer, Martijn van Hasselt, RTI International, Research Triangle Park, North Carolina.

## Abstract

**Introduction:**

Smoke-free air laws in restaurants and bars protect patrons and workers from involuntary exposure to secondhand smoke, but owners often express concern that such laws will harm their businesses. The primary objective of this study was to estimate the association between local smoke-free air laws and economic outcomes in restaurants and bars in 8 states without statewide smoke-free air laws: Alabama, Indiana, Kentucky, Mississippi, Missouri, South Carolina, Texas, and West Virginia. A secondary objective was to examine the economic impact of a 2010 statewide smoke-free restaurant and bar law in North Carolina.

**Methods:**

Using quarterly data from 2000 through 2010, we estimated dynamic panel data models for employment and sales in restaurants and bars. The models controlled for smoke-free laws, general economic activity, cigarette sales, and seasonality. We included data from 216 smoke-free cities and counties in the analysis. During the study period, only North Carolina had a statewide law banning smoking in restaurants or bars. Separate models were estimated for each state.

**Results:**

In West Virginia, smoke-free laws were associated with a significant increase of approximately 1% in restaurant employment. In the remaining 8 states, we found no significant association between smoke-free laws and employment or sales in restaurants and bars.

**Conclusion:**

Results suggest that smoke-free laws did not have an adverse economic impact on restaurants or bars in any of the states studied; they provided a small economic benefit in 1 state. On the basis of these findings, we would not expect a statewide smoke-free law in Alabama, Indiana, Kentucky, Missouri, Mississippi, South Carolina, Texas, or West Virginia to have an adverse economic impact on restaurants or bars in those states.

## Introduction

A total of 29 states and Washington, DC, have laws that prohibit smoking in restaurants and bars (1). Most remaining states without statewide smoke-free laws are home to many cities and counties that have local laws requiring restaurants or bars to be 100% smoke-free. In many of these states, momentum is building to extend the protection offered by local smoke-free laws to all citizens. However, owners of restaurants and bars are concerned that laws prohibiting smoking in their establishments will hurt business. Opponents of smoke-free laws argue that smoke-free policies decrease the number of customers that go to restaurants and bars or the frequency with which they visit those establishments, thus reducing revenue and ultimately, employment.

Research in the past 2 decades has provided clear evidence that smoke-free laws have no adverse effects on the economic performance of restaurants or bars (2–22). Continued expansion of smoke-free laws in the United States would benefit from additional studies demonstrating neutral or even positive effects of such laws on the hospitality industry (2). The primary objective of this study was to estimate the association between local smoke-free air laws and economic outcomes in restaurants and bars in 8 states without statewide smoke-free air laws to obtain information about the likely economic impact of a statewide smoke-free air law in the selected states. The 8 states studied were Alabama, Indiana, Kentucky, Mississippi, Missouri, South Carolina, Texas, and West Virginia. A secondary objective of this study was to examine the economic impact of a 2010 statewide smoke-free restaurant and bar law in North Carolina.

## Methods

### Study design

We estimated dynamic panel data regression models, which used the variation in the presence and restrictiveness of smoke-free air laws over time and across communities, to estimate the average effect of these laws on restaurants and bars in each of the 9 states from 2000 through 2010; we used quarterly data in these calculations. We estimated models for each state separately. Restaurant and bar employment were county-level dependent variables, whereas data on per capita sales outcomes were available at the city level. For the county models, we combined data from all counties for which data were available, whether smoke-free or not, and compared the average effect of smoke-free laws in counties that contain smoke-free communities with counties that have no smoke-free communities. For the city models, we pooled data from all cities with smoke-free laws for which data were available and estimated the average effect of smoke-free laws in those communities.

### Selection of study communities

Nine states were included in the study: Alabama, Indiana, Kentucky, Mississippi, Missouri, North Carolina, South Carolina, Texas, and West Virginia. Because our main objective was to assess the likely economic impact of a hypothetical statewide smoke-free law, we chose states that did not have a statewide law at the time of our study; because the Southeast has generally been more resistant to state smoke-free laws, we decided to focus on this region. Thus, Alabama, Indiana, Kentucky, Mississippi, Missouri, South Carolina, Texas, and West Virginia were chosen for 3 reasons: 1) none had a statewide law that prohibited smoking in either restaurants or bars when we conducted our analysis (Indiana adopted a statewide smoke-free law prohibiting smoking in most workplaces, including restaurants but not bars, on July 1, 2012); 2) all had many communities in which local laws prohibited smoking in restaurants and bars; and 3) all were located in or adjacent to the Southeast. North Carolina was included as an example of a southeastern state that had adopted a statewide smoke-free law. North Carolina’s statewide law on smoke-free restaurants and bars went into effect on January 2, 2010. Before then, no North Carolina community had a smoke-free law.

In the selected states, we identified communities with 100% smoke-free laws in restaurants or bars that went into effect during 2000 through 2010 by using a list of smoke-free communities compiled by the American Nonsmokers’ Rights Foundation (23). We identified 254 cities or counties that had laws on smoke-free restaurants or bars; 216 were included in the study. Thirty-eight were excluded because of incomplete or unavailable data.

### Economic outcome variables

We used 3 economic outcomes as dependent variables: 1) number of restaurant employees at the county level, 2) number of bar employees at the county level, and 3) restaurant and bar sales at the city level. Quarterly employment data for counties in all 9 states were obtained from the US Bureau of Labor Statistics’ Quarterly Census of Employment and Wages (24) for North American Industrial Classification System (NAICS) codes 7221 (full-service restaurants) and 7224 (drinking establishments). Employment data were not available for all counties. We obtained city-level sales data for smoke-free cities in Missouri and Texas. In Missouri, city sales data were provided for “eating and drinking places” (Standard Industrial Classification [SIC] code 58 from the Missouri Department of Revenue). In Texas, sales data were provided by the Texas Comptroller of Public Accounts; we used the same NAICS codes for city-level data on restaurants and bars as we used for county-level data.

### Measurement of smoke-free laws

For the county-level models of restaurant and bar employment in Alabama, Indiana, Kentucky, Mississippi, Missouri, South Carolina, and Texas, we measured smoke-free laws by the percentage of a county’s population that was covered by a smoke-free restaurant or bar law. The regression coefficient for this variable represents the number of restaurant or bar jobs gained or lost for each additional percentage-point of the population that is covered by the smoke-free law. For the county-level models of restaurant and bar employment in North Carolina (which had a statewide law) and West Virginia (which had all county-level laws), we measured smoke-free laws by an indicator variable equal to zero in all time periods preceding implementation of the law and equal to 1 in the time period in which the law took effect and all subsequent periods. The regression coefficient for this variable represents the number of restaurant or bar jobs gained or lost after implementation of the smoke-free law. For the city-level models of restaurant and bar sales, we measured smoke-free laws by an indicator variable equal to zero in all time periods preceding implementation of the law and equal to 1 in the time period in which the law took effect and all subsequent periods. The regression coefficient for this variable represents the change in per capita sales after implementation of the smoke-free law.

### Control variables

Employment and sales in restaurants and bars exhibited a high degree of correlation between past and present values. To account for the dynamic nature of employment and sales, we included the lagged value from the previous calendar quarter as a control variable.

It is important to control for general economic activity and conditions that may affect restaurants and bars, independent of the implementation of smoke-free laws. We accomplished this in 2 ways. First, we included a variable for nonsector employment or sales in each model. For models of restaurant or bar employment, “nonsector employment” is the difference between total employment in all industries and employment in restaurants or bars. For models of restaurant or bar sales (or both), “nonsector sales” is the difference between total sales and sales in restaurants and/or bars. “Total sales” refers to sales data obtained from holders of sales or use-tax permits. We did not include sales from businesses that sell only goods that are outside the sales tax base. In general, sales and use taxes are imposed on all retail sales, leases and rentals of most goods, and taxable services. Second, seasonal effects, such as summer or winter tourism, may affect restaurant or bar employment and sales at regular intervals year after year. To account for these effects, we included quarterly seasonal indicator variables in all models.

We also included the annual number of tax-paid per-capita cigarette sales in each state from *The Tax Burden on Tobacco* (25) to account for potential confounding due to variation in smoking rates. Finally, we controlled for unmeasured differences between counties or cities by including a set of county or city indicator variables.

### Statistical analysis

We estimated all employment models and sales models in Missouri by using the ivreg2 command (26) in Stata version 11 (StataCorp LP, College Station, Texas) (27), which estimates a single equation model by using a 2-step feasible generalized methods-of-moments estimator. This estimator is an instrumental variables (IV) estimator, which we used because the nonsector employment and nonsector sales control variables were endogenous. That is, it was likely that unobserved factors simultaneously affected both the outcome variable and the nonsector employment control variable. Failure to account for endogeneity would lead to bias in the ordinary least squares (OLS) regression estimates. The estimator we used was based on identifying a variable (the “instrument”) for each endogenous control, such that it was related to the control but unrelated to remaining unobserved factors. In our study, the chosen instruments were lagged values of either nonsector employment or nonsector sales. To account for the possibility that the regression errors were correlated over time, we calculated standard errors that are robust to both heteroscedasticity and serial correlation of the residuals. In the Texas sales models, the IV estimator failed the weak instrument test (Kleibergen and Paap’s rank statistic [28] via the first-stage *F* statistic); we therefore used an OLS estimator instead.

## Results

In all states except West Virginia, we found no significant association between smoke-free restaurant laws and restaurant employment (Table 1). In West Virginia, we found a significant increase in restaurant employment in smoke-free counties compared with counties that were not smoke-free. 

**Table 1:** Regression Results^a^ for County-Level Restaurant Employment, Study on Economic Impact of Smoke-Free Laws in 9 States, 2000–2010

Independent Variable	Alabama	Indiana	Kentucky	Mississippi	Missouri	North Carolina	South Carolina	Texas	West Virginia
Smoke-free law^b^	0.59 (0.51)	0.10 (0.25)	0.37 (0.27)	−0.11 (0.14)	0.37 (0.40)	−3.09 (12.25)	0.09 (0.52)	0.34 (0.30)	5.49^c^ (2.19)
Lagged restaurant employment^d^	0.70^c^ (0.04)	0.90^c^ (0.02)	0.84^c^ (0.04)	0.91^c^ (0.02)	0.81^c^ (0.03)	0.68^c^ (0.05)	0.57^c^ (0.05)	0.93^c^ (0.02)	0.92^c^ (0.02)
Nonrestaurant employment	63.21^c^ (17.34)	−23.34 (17.82)	−83.58 (46.73)	40.32 (25.04)	1.92 (21.31)	154.22^c^ (29.92)	155.50^c^ (36.90)	27.65 (17.50)	11.54 (30.93)
Annual state per capita cigarette sales	−2.33^c^ (0.46)	−0.21^c^ (0.07)	−0.01 (0.09)	−0.24 (0.23)	−1.24^c^ (0.36)	−1.92^c^ (0.39)	−9.44^c^ (1.79)	−0.59^c^ (0.22)	0.07 (0.19)
Total no. of observations	1,540	3,555	1,725	1,418	3,334	3,011	1,296	4,525	1,511
Total no. of counties included in analysis	40	85	47	37	86	78	32	117	38
No. of counties with smoke-free restaurant laws included in analysis	15	18	12	16	7	78	12	23	22

a All models include indicators for season and county. Robust standard errors indicated in parentheses.

b The smoke-free law variable is coded as the percentage of the population that is covered by a smoke-free restaurant law for Alabama, Indiana, Kentucky, Mississippi, Missouri, South Carolina, and Texas. In North Carolina and West Virginia, the smoke-free law variable is coded as zero before implementation of the law and as 1 afterward.

c *P* < .05.

d Previous quarter’s restaurant employment.

The estimated coefficient of 5.49 (Table 1) implies an increase of 5.49 restaurant jobs after implementation of a county-wide smoke-free law. Each county in West Virginia that adopted a smoke-free law in restaurants had an average of 527 restaurant jobs before the law. Therefore, smoke-free restaurant laws in West Virginia were associated with an average increase of about 1% in restaurant jobs per county. The first-stage *F* statistics indicated that the instrument (lagged nonrestaurant employment) was strongly related to the endogenous variable (nonrestaurant employment). Among the 9 states, the lowest *F* statistic value (*F* = 208) was for Kentucky, which far exceeds the rule-of-thumb threshold of 10 that is commonly used (29).

In all models, lagged restaurant employment was significant, suggesting that employment in restaurants was highly correlated over time. The coefficients of lagged restaurant employment indicate that employment was moderately (South Carolina, coefficient 0.57) to highly (Texas, coefficient 0.93) persistent from quarter to quarter. Nonrestaurant employment was significant and positive in 3 states: Alabama, North Carolina, and South Carolina. Per capita cigarette sales was significant and negative in 6 of 9 states, suggesting that states with greater amounts of smoking have fewer restaurant jobs on average.

Similar to the results for restaurant employment, lagged bar employment was significant and positive, indicating that bar employment was moderately (South Carolina, 0.62) to highly (Texas, 0.92) persistent from quarter to quarter(Table 2). Nonbar employment was significant but positive in only 2 states, Alabama and Missouri. Annual per capita cigarette sales were significant and negative only in North Carolina. The first-stage *F* statistics again indicated that the instrument was not weak; the minimum value was 182 for Mississippi. We found no significant association between smoke-free bar laws and bar employment in any state.

**Table 2:** Regression Results^a^ for County-Level Bar Employment, Study on Economic Impact of Smoke-Free Laws in 9 States, 2000–2010

Independent Variable	Alabama	Indiana	Kentucky	Mississippi	Missouri	North Carolina	South Carolina	Texas	West Virginia
Smoke-free law^b^	0.02 (0.06)	0 (0.07)	0 (0.09)	0.06 (0.05)	0.03 (0.14)	2.24 (5.54)	−0.08 (0.09)	0.01 (0.11)	−9.97 (5.55)
Lagged bar employment^c^	0.82^d^ (0.04)	0.89^d^ (0.02)	0.83^d^ (0.03)	0.74^d^ (0.06)	0.82^d^ (0.03)	0.85^d^ (0.03)	0.62^d^ (0.06)	0.92^d^ (0.04)	0.82^d^ (0.06)
Non-bar employment	2.52^d^ (1.21)	1.68 (2.13)	−9.07 (6.87)	−8.03 (6.62)	3.97^d^ (1.92)	2.71 (2.47)	−8.28 (4.89)	2.37 (1.25)	12.37 (10.78)
Annual state per capita cigarette sales	0.01 (0.17)	0.06 (0.05)	0.15 (0.09)	0.39 (0.23)	0.79^d^ (0.22)	−0.20^d^ (0.10)	−0.58 (0.49)	0.08 (0.21)	0.06 (0.17)
Total no. of observations	414	940	263	154	683	736	482	1,102	274
Total no. of counties included in analysis	11	25	7	4	17	19	12	28	8
No. of counties with smoke-free bar laws included in analysis	6	7	3	2	4	19	9	14	2

a All models include indicators for season and county. Robust standard errors indicated in parentheses.

b The smoke-free law variable is coded as the percentage of the population that is covered by a smoke-free bar law for Alabama, Indiana, Kentucky, Mississippi, Missouri, South Carolina, and Texas. In North Carolina and West Virginia, the smoke-free law variable is coded as zero before implementation of the law and as 1 afterward.

c Previous quarter’s bar employment.

d *P* < .05.

The first-stage *F* statistics in the Texas models were low: 1.59 in the restaurant model and 0.06 in the bar model. We found no qualitative differences between the OLS and IV estimates, however. In Missouri and Texas, implementation of a smoke-free air law for bars or restaurants (or both) was not significantly associated with a change in per capita sales (Table 3). In all 3 sales models, per capita sales in the previous period were a significant predictor of per capita sales in the current period.

**Table 3:** Regression Results^a^ for City-Level Per Capita Restaurant and Bar Sales in Missouri and Texas, Study on Economic Impact of Smoke-Free Laws in 9 States^b^, 2000–2010

Independent Variable	Missouri Eating and Drinking Establishments^c^	Texas Restaurants^d^	Texas Bars^d^
Indicator for smoke-free restaurant law	−15.97 (20.53)	2.60 (2.66)	—
Indicator for smoke-free bar law	−57.25 (35.38)	—	−0.81 (0.83)
Lagged sector per capita sales^e^	0.28^f^ (0.05)	0.83^f^ (0.03)	0.66^f^ (0.07)
Nonsector per capita sales^g^	−0.10^f^ (0.03)	0 (0)	0^f^ (0)
Annual state per capita cigarette sales	2.43^f^ (1.11)	−0.05 (0.18)	0.10^f^ (0.05)
Number of observations	9,200	1,584	1,266
Number of cities with smoke-free restaurant and/or bar laws included in analysis	14	44	27

a All models include indicators for season and city. Robust standard errors indicated in parentheses.

b The 9 states were Alabama, Indiana, Kentucky, Mississippi, Missouri, North Carolina, South Carolina, Texas, and West Virginia.

c Standard Industrial Classification code 58 for “eating and drinking places.”

d Ordinary least squares estimates for Texas city-level sales models.

e Previous quarter’s sector per capita sales.

f *P* < .05.

g Nonsector sales is the difference between total sales and sales in restaurants or bars (or both).

## Discussion

In this study, we estimated the economic impact of local smoke-free laws in 216 communities in 8 states that did not have statewide smoke-free laws: Alabama, Indiana, Kentucky, Mississippi, Missouri, South Carolina, Texas, and West Virginia. We found no significant association between smoke-free laws and economic outcomes in restaurants and bars in 7 of the 8 states. In West Virginia, restaurant employment increased by a significant 1% after implementation of a smoke-free restaurant law. Based on these findings, we would not expect statewide smoke-free laws to have an adverse economic impact on restaurants or bars in these states. We also examined the association of a statewide smoke-free restaurant and bar law on employment in North Carolina. We found no evidence that North Carolina’s statewide law had affected restaurant or bar employment. This result is consistent with a study that found no impact from North Carolina’s smoke-free law on gross revenues in restaurants or bars (30). 

Our findings are consistent with previous studies (2–22) and the conclusions of the US Surgeon General (31), all of which indicate that smoke-free laws do not negatively impact restaurant and bar business. More importantly, smoke-free laws improve both employee and population health. Indeed, averting the adverse health consequences of secondhand smoke exposure among nonsmoking adults and children is the primary goal of any smoke-free policy. Comprehensive smoke-free laws that completely eliminate smoking in indoor public places and workplaces, including restaurants and bars, have been shown to reduce secondhand smoke exposure among nonsmoking hospitality workers (31) and the general population of nonsmokers (32). Such laws have also been shown to reduce sensory and respiratory symptoms and improve lung function in nonsmoking hospitality workers (19), help workers who smoke to quit (31), and may reduce smoking initiation among youth (33).

A strength of this study is that it was based on data from 216 cities and counties and 9 states during an 11-year period; it is the largest economic impact study of smoke-free laws to date. The panel model estimation approach takes advantage of variation across communities over time and controls for general economic activity, tax-paid cigarette sales, seasonality, endogeneity, and autocorrelation. However, it is unlikely that we accounted for every factor that might have affected the restaurant and bar industries in each state. Nonetheless, the consistency of the results across states strengthens the conclusion that smoke-free laws have not had an adverse economic impact on employment and sales in restaurants and bars.

A limitation of this study is that sales data were available for far fewer states and cities or counties than employment data, especially for bars. Additionally, employment data were missing for many counties in each state, which limits the generalizability of the results, particularly for bars. This analysis, like many previous analyses, examined the average economic impact of smoke-free laws on restaurants and bars in an area and did not assess the economic effects of these laws on individual establishments. Finally, the models did not control for spill-over effects either between adjacent communities or between restaurants and bars (2). Spill-over effects may be relevant in communities that require restaurants but not bars to be smoke-free.

Consistent with similar studies, this study found no significant adverse economic effects on restaurants or bars from laws prohibiting smoking in those venues. At the time of this writing, Alabama, Kentucky, Mississippi, Missouri, South Carolina, Texas, and West Virginia did not have statewide laws banning smoking in restaurants and bars; Indiana enacted a statewide law prohibiting smoking in most workplaces, including restaurants but not bars, on July 1, 2012. On the basis of our results, we would not expect restaurants and bars in these states to experience adverse economic consequences should such a statewide smoke-free law be passed. Rather, all citizens would enjoy the health benefits of being protected from exposure to secondhand smoke while patronizing or working in restaurants and bars.
